# Sero-epidemiological assessment of *Chlamydia trachomatis* infection and sub-fertility in Samoan women

**DOI:** 10.1186/s12879-016-1508-0

**Published:** 2016-04-21

**Authors:** S. Menon, S. H. Stansfield, M. Walsh, E. Hope, L. Isaia, A. A. Righarts, T. Niupulusu, S. V. A. Temese, L. Iosefa-Siitia, L. Auvaa, S. A. Tapelu, M. F. Motu, T. Suaalii-Sauni, P. Timms, P. C. Hill, W. M. Huston

**Affiliations:** Institute of Health and Biomedical Innovation, Queensland University of Technology, Brisbane, Australia; Centre for International Health, University of Otago, Dunedin, New Zealand; National University of Samoa, Apia, Samoa; National Health Service Laboratory Division, Apia, Samoa; Samoa Family Health Association, Apia, Samoa; Centre for Samoan Studies, National University of Samoa, Apia, Samoa; Samoa National Council of Churches, Apia, Samoa; Samoa AIDS Foundation, Apia, Samoa; Victoria University of Wellington, Wellington, New Zealand; Faculty of Science, Health, Education and Engineering, University of the Sunshine Coast, Maroochydore, Australia; School of Life Sciences, Faculty of Science, University of Technology Sydney, Broadway, Sydney, NSW Australia; School of Life Sciences, University of Technology Sydney, PO BOX 123, Broadway, Sydney, NSW 2007 Australia

**Keywords:** Chlamydia, Pacific islands, samoa, female sub-fertility, diagnosis, serology

## Abstract

**Background:**

In our recent village-based cross-sectional study, the prevalence of nucleic acid amplification technique (NAAT) diagnosed *Chlamydia trachomatis* (CT) in sexually active Samoan women was very high (36 %), and test positivity was associated with sub-fertility. We conducted a serological and epidemiological analysis in these participants to identify if serological data can provide further insight into the potential contribution of CT to sub-fertility in this population.

**Methods:**

Serological prediction of CT associated sub-fertility was conducted using a series of commercial tests. The correlation between fertility or sub-fertility, behavioral factors, and serologically predicted CT associated sub-fertility was determined.

**Results:**

A positive antibody reaction against the *Chlamydia* Major Outer Membrane Protein (MOMP) was significantly associated with sub-fertility, with 50 % of infertile women being positive. Serum IgG and IgA antibodies against MOMP correlated with current infection measured by urine NAAT, suggesting longer term infections are common in this population. *Chlamydia pneumoniae* antibodies were frequently detected in this population (84 %), and unexpectedly, were significantly associated with sub-fertility.

**Conclusions:**

The high prevalence of chlamydial infection and of positive chlamydial sub-fertility results suggests that CT is an important and frequent contributory factor to sub-fertility in this population.

## Background

*Chlamydia trachomatis* (CT) is the most common bacterial sexually transmitted infection (STI) in the world. The infection can result in the development of serious sequelae such as pelvic inflammatory disease (PID), ectopic pregnancy and tubal factor infertility (TFI) in women. The reported prevalence of CT infection is in the range 1.4–8.7 % when the general population in high income countries is screened [1–3]. The prevalence of CT infection in Samoa was previously estimated by Sullivan et al. [4] to be 30.9 % based on antenatal screening. Similarly, in women who attended antenatal clinics between 2004 and 2005 in the Pacific Islands (Fiji, Kiribati, Samoa, Solomon Islands, Tonga, and Vanuatu), CT prevalence was 26.1 % in women under 25 years old, and 11.9 % in women over 25 [5].

The proportion of infertility attributable to CT in the Samoan population is not known. Such infertility results from tissue damage to the fallopian tubes (tubal factor infertility, TFI) that remains after the active infection is cleared, meaning that diagnosis using nucleic acid amplification tests (NAAT) is not necessarily suitable. There are numerous serological or chlamydia antibody tests (CAT) that have been developed to diagnose CT infertility, that have been validated on cohorts of women with evidence of tubal damage detected by hysterosalpingography or laparoscopy [6–11]. In a meta-analysis of published evaluations of various assays, Broeze and co-workers identified that micro immune-fluorescence (MIF) was the most sensitive, but relatively low in specificity [6]. In the same study the MEDAC and ANIlabsystems enzyme linked immunosorbant assays (ELISA) appeared to most specific, although less sensitive than MIF, to diagnose women with uni or bi-lateral tubal damage detected by surgical or sonographic technologies [6]. However, a proportion of women with infertility and who are serologically positive by CAT have no detectable tubal blockage but still require IVF (*in vitro* fertilization) to conceive, and this could be at least partially due to tubal damage not detectable by the current surgical or sonographic methods [7, 12–14]. In lower and middle income countries (LMIC) studies generally report higher prevalence of CT in infertile or sub-fertile women (39-55 %), although the prevalence of CT infection in fertile women is also generally high [15–17].

We recently reported a high prevalence (36.0 % by NAAT) of CT in Samoan women using community-based screening and survey of sexually active women aged 18–29 years having unprotected sex, and current infection was associated with women who were defined as being sub-fertile [14, 18]. Here, we conducted a serological study to evaluate the prevalence of CT associated sub-fertility in these same women.

## Methods

The study design and sampling protocol has been previously reported [15, 18]. Women (n = 239) were recruited into a cross-sectional study on CT and sub-fertility from the Pacific nation of Samoa during 2011. Participant inclusion criteria were age between 18 and 29 years, living in the village for at least a year and being sexually active without using any forms of contraception (including condoms, birth control pills, or other forms of contraception) for at least a year. Women were excluded if they had a medical condition, or had undergone a procedure that made it impossible to become pregnant. Participants provided informed written consent, completed an interviewer-led questionnaire and provided biological samples. The nurse who conducted the interview asked the sexual behavioral questions using socially acceptable language and used a two step approach to gauge sexual behavior (as previously described) [18]. The questionnaire responses were used to assign women to ‘sub-fertile’ (or otherwise ‘fertile’). Sub-fertility was defined as at least 12 months of unprotected intercourse without conceiving a pregnancy [18]. The NAAT results have been previously analysed and presented [18], all participants provided a urine specimen that was analysed for CT infection status using the BD ProbeTec ET assay in accordance with the manufacturer’s instructions and using positive and negative controls (BD Biosciences, USA) [18].

The participant sera were tested for CT antibodies using the following commercial ELISAs: CT-IgG ELISA-plus MEDAC (peptides from the MOMP protein, referred to as MEDAC MOMP, used to diagnose past or current infection), cHSP60-IgG ELISA MEDAC (cSHP60 protein), ANIlabsystems CT IgG (peptides from MOMP, marketed to diagnose CT infertility), CT IgA ELISA MEDAC (used to diagnose current CT infection), *Chlamydia pneumoniae* (CP)-IgG-ELISA MEDAC (used to diagnose current CP infection) (summarized in Table 1). The assay positive or negative results in accordance with the manufacturer’s instructions were used for this study (positive, negative, unequivocal (excluded), invalid (excluded)). Titres were not included in this study as they are not part of these commercial tests. All sera were tested with all assays, however, any that were unequivocal or invalid more than once were excluded from the data for that assay and any participants that did not have a complete dataset and valid test result in every assay were completely excluded from the analysis in Table 2 and Fig. 2. The serological testing of CT associated sub-fertility is difficult because the gold standard (MIF) has low specificity leading to high false positives (although it had the highest accuracy using area under the curve), but is reported to be highly subjective and labor intensive [6]. Therefore we chose to test the population using ELISA as we prioritised highest specificity in order to indicate the amount of CT associated sub-fertility in Samoa, and a format that could enable us to compare several tests in a timely manner. We selected multiple ELISAs to compare the different IgG responses and we also included IgA to indicate how many of the infections were recent. Finally, we included a *Chlamydia pneumoniae* ELISA to enable comparison of the sero-prevalence of a related and very common pathogen.

**Table 1:** Commercial serological assays and previously reported sensitivity and specificity

Assay name	Antigen	Study reference name	Sensitivity	Specificity	Detected group	Ref
MEDACCT-IgG ELISA-plus MEDAC	MOMP	MEDAC MOMP	55 %	87 %	Women with infertility (n = 315)	[7]
cHSP60-IgG ELISA MEDAC	cHSP60	MEDAC cHSP60	69 %	93 %	Women with TFI (n = 70)	[22]
MEDAC IgG CT pELISA and cHSP60 IgG ELISA	Combination of both assays	MEDAC Infertile				[22]
ANIlabsystems CT IgG	MOMP peptides	ANIlab	91 %	84 %	CT NAAT diagnosed infection and infertility (*n* = 303)	[16]
CT IgA ELISA MEDAC	MOMP peptides	MEDAC IgA			NAAT diagnosed current infection	[23]
*C. pneumoniae*-IgG-ELISA MEDAC	MOMP peptides	MEDAC Cpn	91.3 %	93.3 %	Current pneumonia and MIF positive status for *C. pneumoniae*	[24]

**Table 2:** Participant data included/excluded in the Table 3 analysis

Variable	Included, n	Excluded, n	OR (95 % C.I.)	*P* value for OR
Age				
18–24	97	48	1	
25–29	64	29	1.11 (0.63–1.94)	0.720
Smoking				
Never	129	59	1	
Ex-smoker/current	33	18	0.84 (0.44–1.61)	0.600
Fertility status				
Sub-fertile	59	28	1	
Fertile	103	49	1.00 (0.57–1.75)	0.990

Comparison of Age, smoking and fertility status for participants included or excluded in the study. Participants excluded had no statistical difference in age, smoking or fertility status (*P* > 0.05)

All statistical analyses were conducted in R statistical environment (3.0.3) using the ‘EpiR (0.9-57) and 'metafor' package (1.9-2) for calculation and presentation (forest plots) of odds ratios (OR). All analyses were conducted to measure the association of assay results with sub-fertility. OR and 95 % confidence intervals (CI) were calculated with restricted maximum likelihood estimates of error.

## Results

### Antibody test results for CT associated sub-fertility

**Fig. 1:**
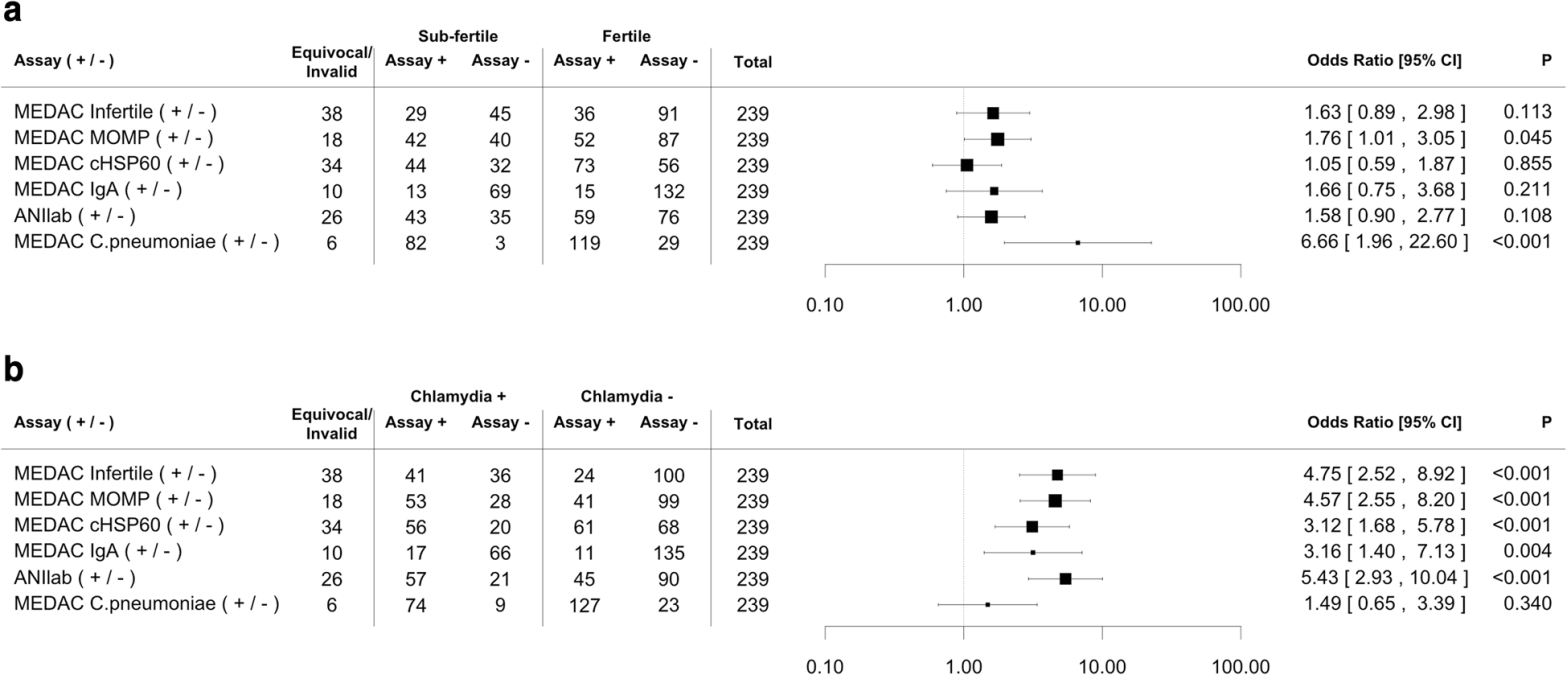
Analysis of the association of serological responses to *Chlamydia* with fertility and CT infection status. **a**. The figure shows Forest plots and Odds Ratio of the association of a positive reaction in the serological assay listed to the right with being infertile. The assay is indicated in the left column. The number of participants that were positive or negative in the serum assay(s) according to their fertile or infertile status are shown on the figure. All 239 participant specimens were tested in each assay. Samples that were unequivocal or not reproducible upon multiple testing were excluded for each assay and these are indicated in the column titled invalid/unequivocal on the table. **b.** The number of participants positive or negative in each serological assay, grouped according to also being positive or negative for current CT infection by urine NAAT (Chlamydia +/−) are shown on the figure. OR with 95 % CI and P values are indicated at the right of the figures

Two hundred thirty-nine women meeting the inclusion and exclusion criteria participated in the study; 90 were defined as being sub-fertile and 149 were defined as fertile with a history of having had a pregnancy. The association of CT with sub-fertility was analysed using each of the commercial serological assays. As shown in Fig. 1a, women who were sub-fertile were significantly more likely to have a positive serological reaction in the MEDAC MOMP assay (p = 0.045). 42 out of 82 sub-fertile women were positive in the MEDAC MOMP assay, whereas 52 out of a total of 139 fertile women were positive. It is important to note that 34 sub-fertile women have been excluded for this assay from Fig. 1a because they had unequivocal results. One of the most common immunological reactions that was associated with infertility in previous studies [9, 12], an antibody response to cHSP60, was not significantly associated with sub-fertility: the sero-prevalence of antibodies to cHSP60 did not differ based on fertility with 57 % of both infertile and fertile women being positive (44 out of 76 sub-fertile women were positive and 73 out of 129 fertile women were positive). The MEDAC Infertile assay, which is recommended by the manufacturer to be a positive reaction in both the MEDAC MOMP and MEDAC cHSP60 assays, was not significantly associated with sub-fertility in this study (in the infertile group 29 women were positive out of 74 total who had results in the assay, and 36 were positive out of a total of 127 women with reportable results in the fertile group).

The overall IgG sero-positivity to CT in this population was 43 % according to the MEDAC MOMP results, and 50 % according to the ANIlab assay. The numbers reported in Fig. 1 vary depending on the number of women that had consistent reportable results in the assay (i.e. we did not include unequivocal or invalid results, these are indicated to the left of the figure).

We included testing for a common respiratory pathogen (*Chlamydia pneumoniae*) to provide an indication of serological responses in our population tested. Women who were sub-fertile were significantly more likely to have a positive reaction in MEDAC CP assay (p < 0.001) (Fig. 1a). The overall presence of sero-positivity to this pathogen (CP) was 86 %. A positive urine NAAT result did not correlate with a positive CP serological result, supporting that this assay is not likely to be detecting CT cross reactivity. Although, pairwise analysis did show that MEDAC MOMP positivity significantly correlated with MEDAC CP (chisel p = 0.042), meaning that women positive in MEDAC MOMP were more likely to be positive in MEDAC CP (OR 3.63 [95 % CI: 0.92–21.11]).

We previously reported that the current infection by urine NAAT was 36 % in this cohort [18], therefore, we evaluated if the serological results correlate with current infection status. Women who were NAAT positive were significantly more likely to be positive in the serological assays for CT by all of assays tested (Fig. 1b). The high association of IgG against MOMP and cHSP60 and current infection by urine NAAT in this cohort could imply these are longer term infections, consistent with the lack of STI screening and treatment programs in this country. A positive result in the MEDAC IgA significantly correlated with NAAT positive status (Fig. 1b) (p = 0.004). This higher frequency of IgG (compared to IgA) correlating with NAAT diagnosed current infection further supports the possibility that in this population there are frequent repeat or longer term infections, given that IgA tends to be produced early and in primary infections.

In the evaluation of demographic factors in relation to subfertility and serological results, we only included data for participants that had a recordable result in every test and answered every demographic question (n = 162). The details of the participants excluded from this analysis are provided in Table 2, and there were no significant differences in the sub-fertility status or other factors in those excluded compared to those included. Age (older women were more likely to be fertile, p < 0.010), MEDAC MOMP (p = 0.040), NAAT positive for CT (p = 0.003) and MEDAC CP (p = 0.007) were all significant factors that associated with sub-fertility in this subset of participants (Table 3).

**Table 3:** Analysis of demographic factors and serological results that associate with sub-fertility

Variable	Sub-fertile, n	Fertile, n2^a^	OR (95 % C.I.)	*P* value for OR
Age				
18–24	47	50	1	
25–29	12	53	0.24 (0.11–0.51)	<0.01
BMI				
Normal Weight	12	21	1	
Overweight	27	35	0.74 (0.31–1.80)	0.498
Obese	20	47	0.74 (0.31–1.80)	0.512
Smoking				
Never	46	83	1	
Ex-smoker/current	13	20	1.17 (0.53–2.57)	0.691
MEDAC Infertile				
Negative	34	72	1	
Positive	25	31	1.71 (0.88–3.33)	0.115
MEDAC MOMP				
Negative	28	66	1	
Positive	31	37	1.97 (1.03–3.78)	0.040
MEDAC cHSP60				
Negative	25	41	1	
Positive	34	62	0.9 (0.47-1.72)	0.749
MEDAC IgA				
Negative	49	89	1	
Positive	10	14	1.3 (0.55-3.14)	0.563
ANIlab				
Negative	29	59	1	
Positive	30	44	1.39 (0.73–2.64)	0.318
MEDAC CP				
Negative	2	22	1	
Positive	57	81	7.74 (1.75–34.21	0.007
CT Infection (NAAT)				
Negative	28	73	1	
Positive	31	30	2.69 (1.39–5.24)	0.003

^a^This table only includes women who had consistent results in all assays (*n* = 162), 59 sub-fertile and 103 fertile women

**Fig. 2:**
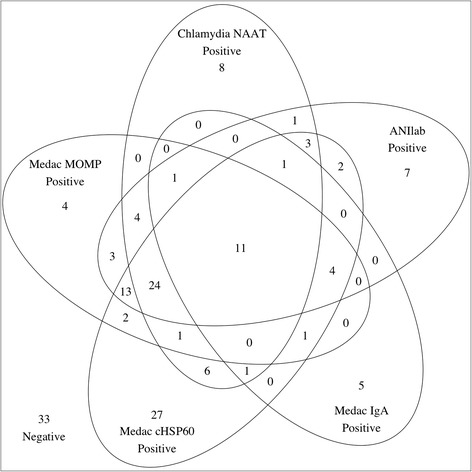
Venn diagram to demonstrate concordance between serological assays and CT NAAT results. The diagram shows the number of participants positive in each of the assays and those who were positive in more than one assay. The two MOMP assays (MEDAC IgG and ANIlab) and NAAT results showed considerable concordance. The samples that were negative in all assays are also indicated on the figure, only the 162 samples that had a valid result in all of these assays are included in the Venn diagram

We used the same subset of specimens to assess test concordance. As shown in the Venn diagram (Fig. 2) there was often concordance between the serological assays and NAAT results, particularly between NAAT, MEDAC MOMP, and ANIlab. This supports the notion that these assays are reporting serological responses to CT and are not likely to be a consequence of non-specific reactivity.

## Discussion

The results of this study imply that up to half (51 % infertile women positive for MEDAC MOMP) of women who are sub-fertile in this population could have CT as a cause or contributing factor. To our knowledge this is one of the highest burdens of CT associated sub-fertility reported to date. This is higher than most studies report, even those conducted in fertility clinics, but is consistent with a fertility clinic study in India that found a similar prevalence [15]. This may be a reflection of the absence of routine screening and treatment for CT infections in this population.

A possible limitation of our study is that the serological results may be influenced by a higher number of repeat infections given the high prevalence of infection in this population. These repeat infections may lead to higher positives in these assays which use absorbance thresholds that have been designed on clinically defined infertile cohorts, often in settings with a much lower background prevalence. The MEDAC Infertile assay was not significantly associated with sub-fertility in this study. Nevertheless, the high prevalence of cHSP60 antibodies (one of the components of the MEDAC test) could suggest that chronic infections or sequelae are high in this population. Most studies that found a significant association had TFI as the specified measure for infertility [9]. In this population we have limited knowledge of the other fertility factors as this was a village-based survey in the absence of any gynecological investigations that would normally be conducted in a fertility clinic. In addition it is of course possible, considering the sensitivities of obtaining sexual behavior information that some women were mis-classified with respect to subfertility. The amount of sexual activity was of course not the same for each woman, affecting their individual probabilities of becoming pregnant.

The study findings indicated a higher fertility rate in the older ages (although the inclusion criteria were limited to the most reproductive years). The higher fertility in the older women is not that unexpected and is likely because this study is based on sexually active women who were all within the ideal reproductive age, so the longer the period of sexual activity the more likely the women are to have conceived. Alternatively, this could be explained by an increased desire of these women (25–29 year olds) to achieve pregnancy leading to increased sexual activity.

It was unexpected to find a significant correlation of sero-positivity to *C. pneumoniae* with sub-fertility in this population. It is difficult to determine if this is actually serological cross reactivity (or a genuine association). Serology to *C. pneumoniae,* or the presence of the organism, has been previously found to significantly correlate with various diseases [19–21], but not with sub-fertility. One possible explanation for this finding could be that the infertile women in this population have tissue lesions or adhesions in the fallopian tube that may form a reservoir for the pathogen and perhaps the immune response to this reserve of CP may exacerbate the CT pathology that results in development of tubal sub-fertility. This persistence in the scarring site could explain the high prevalence of CP serology that, whilst significant, is not causal for sub-fertility. Alternatively, this could be a chance result. Firstly, as the prevalence of *Chlamydia pneumoniae* antibodies is very high (in both fertile and infertile women), statistical analysis tends to lead to an overestimation of the odds ratio. Secondly, the number of participants included in the analysis is relatively low (n = 233).

MEDAC MOMP and MEDAC CP were the only assays that significantly correlated with sub-fertility, suggesting that whilst serology is a much more feasible manner to measure possible chlamydial infertility in the developing world setting the current assays may be confounded by the high prevalence, and only MEDAC MOMP may be suitable.

## Conclusions

The high prevalence of *C. trachomatis* infections in Samoa is likely to be leading to high rates of preventable sub-fertility in this population. The results reported here using serological and epidemiological data collection indicate that CT-associated sub-fertility could be a factor for as much as half of the burden of sub-fertility in sexually active women in this reproductive age range in Samoa.

### Ethics approval and consent to participate

Participants provided informed written consent, completed an interviewer-led questionnaire and provided biological samples. The initial ethical approval was from National University of Samoa, Samoa National Health Service Board approval for the use of the Laboratory and staff, and the Samoa Ministry of Women approved the village based survey, and the Samoan Ministry of Health. Ethical approval was previously reported [19] and Queensland University of Technology Human Research Ethics Committee approval was also obtained (approval number 1100000276).

### Consent for publication

Not applicable.

### Availability of data and methods

All data that is not present in the publication will be made available upon request in de-identified format. The questionnaire development and details are previously presented and can be provided upon request.

## Abbreviations

CI: confidence interval
CT: *Chlamydia trachomatis*
MEDAC Cpn: MEDAC *Chlamydia pneumoniae* pELISA
MEDAC MOMP: MEDAC *Chlamydia trachomatis* pELISA
MEDAC cHSP60: MEDAC *C. trachomatis* cHSP60 ELISA
MEDAC Infertile: combined results from MEDAC MOMP and MEDAC cHSP60 to predict infertile/chronic sequelae outcomes from *C. trachomatis* infections as per the manufacturer’s recommendations
OR: odds ratio
NAAT: nucleic acid amplification test
